# Anti-TNF (adalimumab) injection for the treatment of adults with frozen shoulder during the pain predominant stage protocol for a multi-centre, randomised, double blind, parallel group, feasibility trial

**DOI:** 10.3310/nihropenres.13275.1

**Published:** 2022-04-12

**Authors:** Sally Hopewell, Nicola Kenealy, Ruth Knight, Amar Rangan, Susan Dutton, Cynthia Srikesavan, Marc Feldmann, Sarah Lamb, Jagdeep Nanchahal

**Affiliations:** 1 Oxford Clinical Trials Research Unit / Centre for Statistics in Medicine, Nuffield Department of Orthopaedics, Rheumatology and Musculoskeletal Sciences, University of Oxford, Oxford, UK; 2 Kennedy Institute of Rheumatology, Nuffield Department of Orthopaedics, Rheumatology and Musculoskeletal Sciences, University of Oxford, Oxford, UK; 3 James Cook University Hospital, South Tees Hospitals NHS Foundation Trust, Middlesbrough, UK; 4 Hull York Medical School and Department of Health Sciences, University of York, York, UK; 5 Centre for Rehabilitation Research in Oxford, Nuffield Department of Orthopaedics, Rheumatology and Musculoskeletal Sciences, University of Oxford, Oxford, UK; 6 College of Medicine and Health, University of Exeter, Exeter, UK

**Keywords:** Randomised Controlled Trial; Protocol; Frozen Shoulder; Adhesive Capsulitis; Adalimumab; Anti-TNF.

## Abstract

**Objectives:**

The Anti-Freaze-F trial will assess the feasibility of conducting a large randomised controlled trial to assess whether intra-articular injection of anti-TNF (adalimumab) can reduce pain and improve function in people with pain predominant early stage frozen shoulder.

**Methods and analysis:**

We are conducting a multi-centre, randomised feasibility study, with an embedded qualitative sub-study. We will recruit adults ≥18 years with a new episode of shoulder pain attributable to early stage frozen shoulder, recruited from at least five UK NHS musculoskeletal and related physiotherapy services. Participants (n=84) will be randomised (centralised computer generated 1:1 allocation) to receive either: 1) intra-articular injection of anti-TNF (adalimumab 160mg) or 2) placebo injection (saline [0.9% sodium chloride]), both under ultrasound guidance. A second injection of the allocated treatment (adalimumab 80mg) or equivalent volume of placebo will be administered 2–3 weeks later. All participants will receive a physiotherapy advice leaflet providing education and advice about frozen shoulder and pain management. The primary feasibility objectives are: 1) the ability to screen and identify potential participants with pain predominant early stage frozen shoulder; 2) willingness of eligible participants to consent and be randomised to intervention; 3) practicalities of delivering the intervention, including time to first injection and number of participants receiving second injection; 4) standard deviation of the Shoulder Pain and Disability Index (SPADI) score and attrition rate at 3 months from baseline in order to estimate the sample size for a definitive trial. We will also assess follow up rates and viability of patient-reported outcome measures and range of shoulder motion for a definitive trial. Research Ethics Committee approval (REC 21/NE/0214).

**Trial registration number:**

ISRCTN 27075727; EudraCT number: 2021-003509-23; ClinicalTrials.gov NCT05299242.

## Plain language summary

Frozen shoulder is a common condition affecting approximately 9% of people aged 25–64 years. During the early phase the pain is usually unbearable and the later restriction in movement is severely limiting. It occurs when the flexible tissue (capsule) that surrounds the shoulder joint becomes inflamed, thickened and tight. It's not fully understood why this happens but it is more common in people with diabetes or Dupuytren's disease, which causes the fingers to curl into the palm. It can also occur following shoulder injury or surgery. The pain can be very severe and lasts 3–9 months, followed by a 4–12 month period of increasing stiffness, after which the condition usually improves. Frozen shoulder often affects a person’s ability to sleep, carry out everyday activities, and work. Current treatments include rest, painkillers, anti-inflammatories, physiotherapy and steroid injections. If stiffness persists, surgery is sometimes recommended. However, there is no evidence that any of these treatments lead to significant benefit in the long term, with many being ineffective. Steroid injections only help in the short term.

The aim of this study is to find out if it is possible to run a larger trial to test whether an injection of adalimumab can reduce pain and prevent the disease from getting worse, if given during the early painful phase of frozen shoulder. We need to conduct this smaller study first to be sure it’s possible to identify and treat people with early stage frozen shoulder within the current NHS system, before we conduct a much larger trial to find out if this treatment works.

In this study we will include 84 adults with painful early stage frozen shoulder who have not yet received treatment. People will be randomised to receive either an injection of the drug adalimumab or a dummy injection of saline (placebo) directly into the shoulder joint, both guided by ultrasound. All participants will also receive standardized advice on how to manage their shoulder pain. We will assess participants before treatment and three months later. Adalimumab has been used very successfully to treat other inflammatory diseases such as rheumatoid arthritis. This drug has been chosen as the biological processes underlying frozen shoulder are similar to those in Dupuytren’s disease, where we found it helps to stop the cells causing the disease.

## Introduction

Frozen shoulder (adhesive capsulitis) is an extremely painful and debilitating condition and affected individuals struggle with activities of daily living and significant sleep disturbance as a result of severe pain ^1^. The condition is very common, affecting about 9% of people in the UK aged 25–64 years ^2^, and 20% develop the same problem in the other shoulder ^3^. Frozen shoulder may develop as a primary condition or secondarily following surgery or trauma. Up to 30% of patients with diabetes mellitus develop frozen shoulder and the symptoms are more persistent and recalcitrant in this group ^1^.

The classic description of the development of frozen shoulder is of three overlapping phases ^4, 5^. The initial pain predominant inflammatory phase is characterised by constant pain and difficulty sleeping, and lasts between three and nine months. This progresses to a stiffness predominant fibrotic stage with progressive restriction of motion, particularly external rotation and elevation of the shoulder, and impairment of function, and lasts between four and 12 months. The pain changes from being constant to being manifest at the end of range of motion and of reduced intensity. There is a gradual improvement in range of motion and stiffness over a 12–48 month period, although end of range pain may persist. The average duration of the condition is 30 months (range 1 to 3.5 years) ^6^. Full resolution of symptoms does not always occur.

The aetiology of frozen shoulder is poorly understood and consequently there is no consensus on the optimal treatment. The majority of patients with early stage pain predominant frozen shoulder are managed in primary care or at primary care interface musculoskeletal services by physiotherapists and GPs. During this stage, standard treatment consists of rest, advice, analgesics, physiotherapy and corticosteroid injections to address the symptoms.

There is limited efficacy for the treatments currently offered to patients with frozen shoulder. Two Cochrane reviews have concluded that whilst oral steroid or local steroid injections lead to short term benefit in pain and range of motion, the effects are not maintained beyond six weeks ^7– 9^. Other Cochrane reviews concluded that there is no evidence that physiotherapy or ultrasound therapy are beneficial ^10^, and that manual therapy with exercise is less effective than corticosteroid injection in the short term ^11^. These findings are supported by a Health Technology Assessment report which found that, of all treatment options available, the only short-term benefit was from steroid injection in addition to home exercise in patients with symptoms of less than six months ^12^. In addition, manipulation under anaesthesia was found to be no better than home exercise programme ^12^, and the use of arthrographic joint distention with glucocorticoid and saline was no better than sham procedure ^13^. The United Kingdom Frozen Shoulder Trial (UK FROST) compared the effects of physiotherapy plus corticosteroid injection, manipulation under anaesthesia with a steroid injection, and arthroscopic capsular release supplemented with a steroid injection ^9^. None of the treatments were found to be clinically superior. In addition, UK FROST recruited patients from secondary care, and it was unlikely that people in the initial pain predominant inflammatory phase were included.

Our study (Anti-Freaze-F) is designed to specifically target people with early stage pain predominant frozen shoulder. We will assess the feasibility of conducting a large multicentre randomised trial to test whether giving an intra-articular injection of adalimumab (a drug targeting the inflammatory mediator tumour necrosis factor [TNF]), can reduce pain and prevent the disease from getting worse, if given during the early pain predominant stage (i.e., within approximately three months of onset of symptoms). Whilst the pathogenesis of frozen shoulder remains largely unknown, a recent systematic review confirmed the presence of fibrosis and the role of inflammation ^6^. The affected tissues are infiltrated by immune cells, including macrophages, mast cells, T cells, and there are elevated levels of pro-inflammatory cytokines, including TNF, IL-6 and IL-1ß ^14, 15^, with myofibroblasts contributing to the deposition of excessive matrix components that result in fibrosis ^16^. More than 50% of people with frozen shoulder also have Dupuytren’s disease ^17^ and the underlying pathology of frozen shoulder is similar to Dupuytren’s disease ^16, 18^ where our laboratory studies have shown that the myofibroblast phenotype is critically dependent on the local production of low levels of the pro-inflammatory cytokine TNF ^19^. In a dose-ranging proof of concept phase 2a and 2b clinical trial (RIDD trial) we found that local injection of 40mg of adalimumab in 0.4ml directly into Dupuytren’s nodules resulted in downregulation of the myofibroblast phenotype ^20, 21^.

Anti-TNF drugs have a very strong safety profile, having been used in over 10 million people, adalimumab in over five million, and more than 25,000 patients have been recruited to trials of adalimumab, which is approved for nine different disorders. Adalimumab is not currently licenced for use as an intra-articular injection for pain predominant early-stage frozen shoulder and therefore will be used off license for the purposes of this trial. If successful, the Anti-Freaze-F trial could provide evidence that it is feasible to conduct a trial of Anti-TNF for a very common debilitating shoulder condition, avoiding the need for surgery and prolonged physiotherapy, thereby reducing National Health Service (NHS) cost.

### Objectives

The aim of the Anti-Freaze-F trial is to assess the feasibility of conducting a large randomised controlled trial to assess whether an intra-articular injection of adalimumab (anti-TNF) can reduce pain and improve function in people with early stage frozen shoulder.

The primary objectives of this feasibility study are to assess the:

- ability to screen and identify potential participants with pain predominant early stage frozen shoulder;
- willingness of eligible participants to consent and be randomised to intervention;
- practicalities of delivering the intervention, including time to first injection and number of participants receiving second injection;
- standard deviation (SD) of the Shoulder Pain and Disability Index (SPADI) score and attrition rate at 3 months from baseline in order to estimate the sample size for a definitive trial.

Secondary objectives are to assess the follow up rates and viability of patient reported outcome measures and clinician assessed range of shoulder motion at 3 months. Patient reported outcomes include pain and shoulder function, participant assessed range of shoulder motion, psychological factors, sleep disturbance, return to desired activities, global impression of change and health resource use. An embedded qualitative sub-study will be conducted to explore participants’ experiences of being recruited into the trial.

## Methods

### Study design

**Figure 1.**
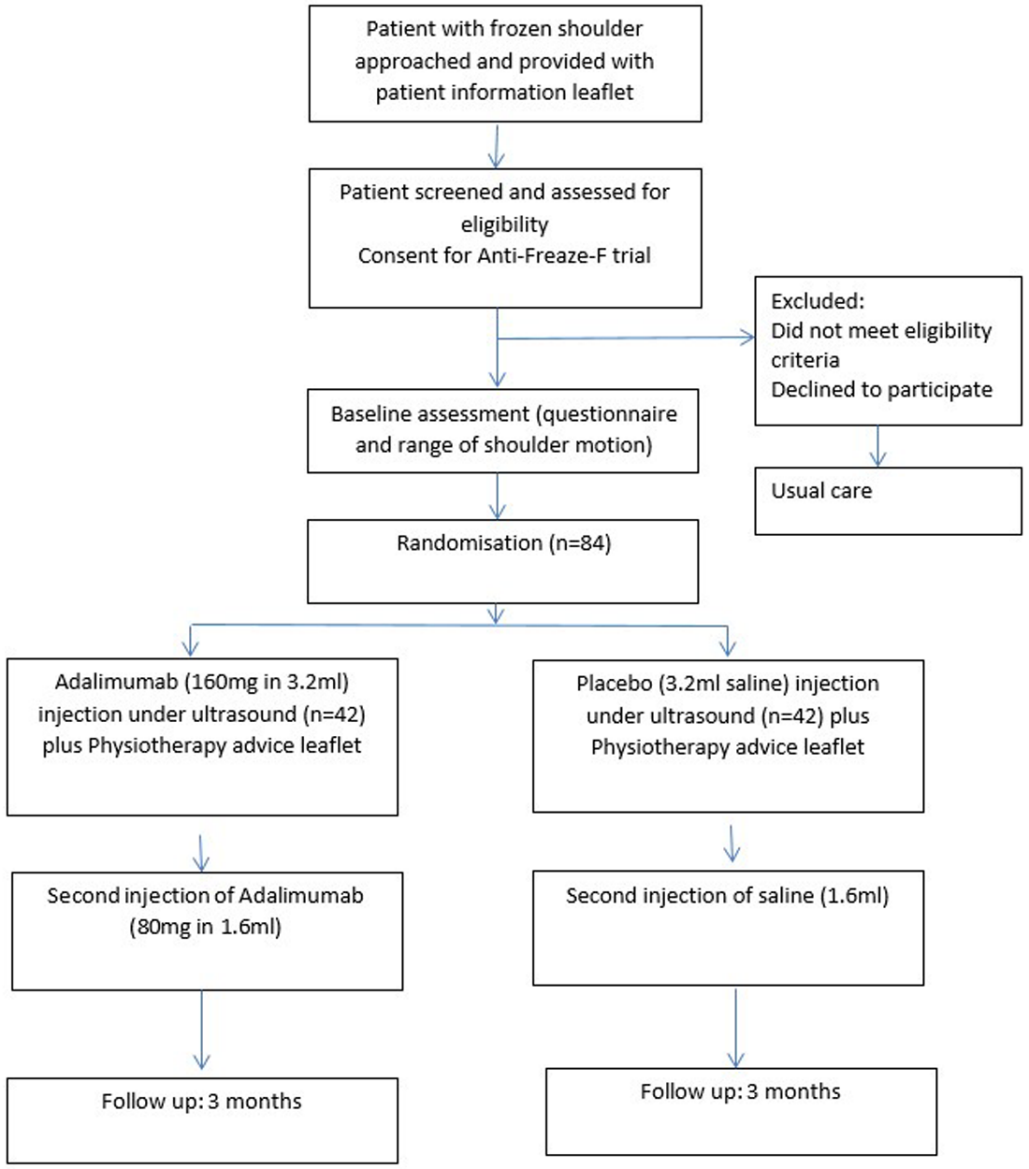
Study flow diagram for Anti-Freaze-F trial.

The trial will be a multi-centre, randomised, double blind, parallel group, feasibility trial, with an embedded qualitative sub-study ( Figure 1).

### Setting

The trial will be conducted across at least five NHS primary and or secondary-care-based musculoskeletal services and their related physiotherapy services, dependant on the local service provision. These services treat people with a range of musculoskeletal conditions and are run by specialist practitioners including extended-scope physiotherapists, general practitioners (GP) with a specialist interest in musculoskeletal conditions, rheumatologists, and orthopaedic surgeons.

### Study participants

Participants with a new episode of shoulder pain attributable to pain predominant stage of frozen shoulder will be recruited from NHS primary-care-based musculoskeletal services and their related physiotherapy services, with treatment delivered within these services or the local secondary care site dependant on the local service provision. Participants may also be recruited from directly from NHS secondary-care-based musculoskeletal services, dependant on local service provision at sites. Imaging, including plain radiographs, may be used to confirm the diagnosis of frozen shoulder and rule out other pathology such as glenohumeral arthritis ^22^ as per standard NHS care (i.e. not as part of trial procedures).

***Eligibility***

Patients will be eligible for this study if they are:

- Men and women aged 18 years and above;
- with a new episode of shoulder pain attributable to pain predominant stage of frozen shoulder (i.e. within approximately three months of onset of symptoms) diagnosed using criteria set out in the British Elbow and Shoulder Society (BESS) guidelines ^23^;
- who are not being considered for surgery;
- able to understand spoken and written English;
- willing and able to give informed consent for trial participation and comply with all study requirements and timeline;
- willing to allow their GP to be notified of participation in the trial;
- if female, and of child-bearing potential and willing to use effective contraception throughout the treatment period and for five months after the last injection.

We will exclude those:

- with frozen shoulder secondary to significant shoulder trauma (e.g., dislocation, fracture or full thickness tear requiring surgery) or other causes (e.g., recent breast cancer surgery or radiotherapy);
- with a neurological disease affecting the shoulder;
- with bilateral early stage frozen shoulder;
- with other shoulder disorders (e.g., inflammatory arthritis, rotator cuff disorders, glenohumeral joint instability) or with red flags consistent with the criteria set out in the BESS guidelines ^23^;
- who have received corticosteroid injection for shoulder pain in the last 12 weeks to either shoulder;
- currently taking any anti-TNF drug, or being treated with coumarin anticoagulants, such as warfarin;
- who have participated in another research study involving an investigational medicinal product in the past 12 weeks;
- with significant renal or hepatic impairment;
- with contra-indications to anti-TNF injection;
- with any other significant disease which, in the opinion of the Investigator, may either put the participants at risk because of participation in the study, or may influence the result of the study.

### Recruitment of participants, screening and eligibility assessment

Potential participants will attend their appointment in accordance with standard NHS procedures at each site. The treating practitioner will undertake a clinical assessment according to their usual practice. If a patient fulfils the criteria for pain predominant stage of frozen shoulder, they will be assessed to see whether they meet the Anti-Freaze-F eligibility criteria. Patients will be provided with a copy of the participant information sheet and asked if they wish to participate in the trial. Those meeting the eligibility criteria and wishing to participate in the trial will be approached for informed consent. Patients who do not meet the eligibility criteria, or who do not wish to participate will receive standard NHS treatment. We will record anonymised information on the age and sex of those who decline to participate so that we can assess the generalisability of those screened. The reasons for declining will be asked and any answers given will be recorded.

### Informed consent, baseline assessment and trial specific screening tests

After participants have been assessed for eligibility, informed consent for participation in the trial will be sought by a research facilitator trained in good clinical practice. The process for seeking, confirming and documenting informed consent will be either paper based (written consent) or digital (e-consent) depending on local facilities at each site. Participants will then be asked to complete the baseline assessment questionnaire that will record demographic information and baseline measurements for the patient reported outcome measures ( Table 1 and Table 2). The questionnaires will be available in both online and paper formats as, due to their frozen shoulder, participants may find one format much easier to complete than the other. Clinician assessed baseline measurement of range of motion of the shoulder will also be performed.

**Table 1.** Outcomes and time points of assessment.

Outcome	Measurement	Time point
Demographic	Age, Sex, Height, Weight, Ethnicity, Smoking, Date of frozen shoulder diagnosis, Duration of symptoms, Hand dominance, Affected shoulder, Diabetes and type, Dupuytren’s Disease	0 months
Shoulder range of movement	Clinician assessed (goniometry measured) active shoulder flexion, extension, abduction, internal and external rotation Patient Reported ROM Questionnaire	0, 3 months
Pain and function	Shoulder Pain and Disability Index (SPADI) ^24, 25^ 13-item total scale	0, 3 months
Pain	Shoulder Pain and Disability Index (SPADI) ^24, 25^ 5-item subscale	0, 3 months
Function	Shoulder Pain and Disability Index (SPADI) ^24, 25^ 8-item subscale	0, 3 months
Psychological factors	Fear Avoidance Belief Questionnaire physical activity 5-item subscale ^26^ Pain Self-efficacy questionnaire (short form) ^27^	0, 3 months
Sleep disturbance	Insomnia Severity Index ^28^	0, 3 months
Global impression of treatment	Patient-rated Likert scale ^29^	3 months
Return to desired activities	Patient-reported return to desired activities, including work, social life and sport activities ^30^	3 months
Health resource use	Consultation with primary and secondary care, additional physiotherapy, injection use, or alternative therapies for index shoulder	3 months
Adverse Events	Any Grade 3 or above AEs that have occurred from Consent up until the 3 month Follow up.	3 months

**Table 2.** Participant timeline.

TIMEPOINT (post randomisation unless stated otherwise)	Screening	0–2 weeks	4–5 weeks	3 months
ENROLLMENT:				
Screening log	✓			
Informed consent	✓			
Eligibility confirmed	✓			
Randomisation	✓			
INTERVENTIONS:				
1 ^st^ Anti-TNF/placebo injection		✓		
Physiotherapy advice leaflet provision	✓			
2 ^nd^ Anti-TNF/placebo injection *			✓	
ASSESSMENTS:				
Serological testing e.g. TB ELISpot, Hepatitis B surface antigen		✓		
Pregnancy testing (if needed)	✓			
Baseline questionnaire	✓			
ROM Assessment	✓			✓
Follow-up questionnaire				✓
Follow-up clinic visit				✓
Qualitative interview (optional) **				✓
AE Reporting (grade 3 or above)	✓	✓	✓	✓

*2 ^nd^ injection must be 2–3 weeks after the 1 ^st^ injection **interview conducted within 4 weeks of intervention delivery

Participants will undergo serological testing to check for latent Tuberculosis (TB) and Hepatitis B surface antigen. Blood tests will be performed during the baseline assessment or at the time the participant attends for their first injection appointment (depending on the local site provision). The risks of reactivation following a single injection are low; any participant for whom the serology test result shows positive will be referred to their local infectious diseases service and will not receive the second injection.

### Randomisation

Consented participants will be randomised to intervention groups (1:1) by the site research facilitator using the centralised randomisation service provided by the Oxford Clinical Trials Research Unit. Randomisation will be computer-generated and stratified by study site using a variable block size to ensure the participants from each study site have an equal chance of receiving either intervention.

### Blinding

Study participants and site staff, except pharmacy staff, will be blinded to treatment allocation. The clinician delivering the treatment injection will not be blinded but will not be involved with any further trial-specific assessment of the participant. The trial statistician and data entry personnel will not be blinded to the treatment allocation. The remaining members of the trial management team, including the staff conducting the qualitative interviews, will be blinded to treatment allocation until after data analysis is complete.

### Interventions

***Adalimumab/Placebo Injection***

Eligible participants will be allocated to receive either an intra-articular injection of adalimumab (160mg in 3.2ml for the first injection, 80mg in 1.6ml for second injection) or placebo (normal saline [0.9% sodium chloride] 3.2ml for the first injection and 1.6ml for the second injection). The dose of 160mg followed by 80mg two weeks later was selected as this is the approved loading dose in patients with inflammatory bowel disease ^31^.

The injection will be given approximately within two weeks of randomisation into the anterior shoulder joint space in the rotator cuff interval where there is maximal inflammation of the capsule and synovium ^32^, under guided ultrasound by an appropriately qualified practitioner. The practitioner will be either a GP with a specialist interest in musculoskeletal conditions, rheumatologist, extended scope physiotherapist, orthopaedic surgeon, sonographer or radiologist, dependant on the local service provision at study sites. The practitioner will confirm the participant is still in the pain predominant phase before administering the first injection. If the participant is no longer in the pain predominant phase, they will not receive the injection and the reason will be recorded.

We will use adalimumab in vials supplied by Fresenius Kabi Ltd (Idacio 40mg/0.8 ml) rather than a pre filled syringe or pen as the needles fitted to the pre-filled syringes are too short for shoulder joint injection. The adalimumab/placebo injection will be dispensed by the local site pharmacy and sealed in identical sized and sealed opaque plastic bags in order to maintaining blinding by staff who are blinded to treatment allocation. Preparation of the adalimumab/placebo injection will take place in a clinic room/area separate from the participant to ensure the participant remains blinded to their treatment allocation immediately prior to injection. Both adalimumab and placebo have a similar viscosity and appearance so the two treatments will be indistinguishable. The same type of syringe and needle will be used for injection of both adalimumab and saline thus maintaining the blinding of the participant and staff not involved in preparation and administration of the injection. The skin at the site of injection of adalimumab/placebo may be infiltrated with local anaesthetic to reduce the pain of the injection in accordance with local practice. Once adalimumab has been drawn up, it tends to lose it potency and this precludes preparation of the syringes before the patient presents for treatment. For this trial, no more than 30 minutes will elapse from when the injection is drawn up from the vials to when the injection is given.

All participants irrespective of whether they are still in the pain predominant phase, will receive a second injection (adalimumab/placebo) administered 2–3 weeks after the first injection, unless the participant declines the second injection, has a related grade 3 or above adverse event after the first injection or tests positive for TB or hepatitis B surface antigen. Injection details, including time from randomisation to injection delivery, will be recorded on a trial specific injection treatment log. Participants will be advised they can continue with their physiotherapy and resume normal day-to-day activities immediately after the injection. Participants will be provided with a written information leaflet advising them that there will be no restriction on their activities after the injection and what to do if they experience any side effects.

***Physiotherapy advice leaflet***

All participants will receive a written physiotherapy advice leaflet providing education and advice about frozen shoulder and pain management ^4^. Physiotherapy during the early pain predominant stage of frozen shoulder is primarily directed at pain relief (e.g., heat, cold and other pain relieving modalities) as forcing the joint to move can make it more painful and is best not pursued. Participants will be advised to take over-the-counter analgesia as required, in accordance with the BESS guidelines ^23^. They will be provided with advice on modifying activities that exacerbate symptoms and on sleeping positions ^33^. The advice leaflet will also include simple self-guided exercises, which participants can use to increase shoulder joint mobilisation, once the early pain predominant stage reduces. Exercises include passive mobilisation of the shoulder and capsular stretching ^9^. Joint mobilisation combined with stretching exercises has been found to be more effective than stretching exercises alone ^34, 35^. As low health literacy levels are a major consideration when developing patient-facing materials, plain English and patient representative involvement will be used to optimise readability.

***Concomitant care***

Participants may seek other forms of treatment during the follow-up period of the trial but will be informed that they should use usual routes (e.g., through GP referral) to do so. Additional treatments, including consultation with their GP or other health professional will be recorded.

### Outcomes

***Feasibility objectives***

The main aim of this feasibility study is to determine whether a future definitive trial would be feasible and to determine the sample size for the definitive trial to assess the effectiveness of anti-TNF adalimumab. We will focus on the main areas of uncertainty relating to the acceptability to be randomised to intra-articular injection of adalimumab and the ability to identify and recruit and treat participants who have pain predominant early stage frozen shoulder within the current NHS patient pathway for musculoskeletal conditions. In addition, we will collect outcome measures at three months, including SPADI and range of shoulder motion. To determine the feasibility of a definitive randomised controlled trial, the success criteria will be:

- Ability to screen and identify potential participants with pain predominant stage frozen shoulder.
- Willingness of eligible participants to consent and be randomised to intervention.
- Practicalities of delivering the intervention, including time to first injection and number of participants receiving second injection.

Data to assess our feasibility objectives will be collected at each site via a trial specific screening log; reasons for ineligibility and/or participants declining to participate in the trial will be recorded where available. Injection details, including time from randomisation to injection delivery, will be monitored based on the information recorded on the trial specific injection treatment logs.

***Outcomes***

Outcomes ( Table 1) will be collected at baseline and at three-months to assess the feasibility of collecting these in a future definitive trial and to obtain the variability estimates required for estimation of the sample size of the definitive trial. Patient reported outcomes will include shoulder pain and function measured using the SPADI scale (primary outcome for definitive trial) ^24, 25^; sub-domains of pain (SPADI 5-item pain subscale), function (SPADI 8-item disability subscale) ^24, 25^; shoulder range of motion (Participant Shoulder Movement Questionnaire); psychological factors (Fear Avoidance Belief Questionnaire) ^36^; pain self-efficacy questionnaire ^37^; sleep disturbance (Insomnia Severity Index) ^38^; patient global impression of change ^29^; return to desired activities; additional health resource use for index shoulder (e.g. consultation with primary and secondary care, additional physiotherapy, injection use, or alternative therapies). The choice of outcome measures is based on Outcome Measures in Rheumatology (OMERACT) 2016 core outcome set for shoulder disorders ^36^ and a systematic review of core outcomes used in studies of frozen shoulder ^37^.

At baseline and three month follow up, a blinded assessor will use a universal manual goniometer to measure range of shoulder movements, including active flexion, extension, abduction internal and external rotation, limitation of which has been shown to be pathognomonic of frozen shoulder in the absence of glenohumeral arthritis ^38^.

### Adverse events

The safety profile of adalimumab is well known, with the most common adverse reactions being mild injection site reactions. The Common Terminology Criteria for Adverse Events (CTCAE) v5.0 will be used to guide recording adverse events including grading of the event. Only clinician assessed adverse events, graded 3 and above, occurring during the trial for each participant, from their consent until the three month follow up and that are considered related to the trial medication (adalimumab/placebo) will be recorded. Participants will be asked by the treating clinician at their second injection visit if they have experienced any adverse events as a result of their first injection. Similarly, participants will be asked if they have experienced any adverse events as a result of their second injection at the three month follow up appointment.

### Follow up data collection

Follow up will be conducted via face-to-face clinic assessment and patient reported questionnaire at three months after randomisation ( Table 1). Participants who are unable to attend the face-to-face appointment will be asked to complete the questionnaire and return it to the Anti-Freaze-F trial office in a prepaid envelope or submit online as appropriate. The reason why a participant is unable to attend the face-to-face clinic appointment will be recorded and whether this was due to potential COVID-19 restrictions or other reasons. For those who do not respond, at least one reminder will be sent. Telephone and email follow-up will also be used to contact those who do not respond to the reminder or who have not fully completed the returned questionnaire.

### Data management

All data will be processed according to the UK General Data Protection Regulations (GDPR) and the Data Protection Act 2018. All documents will be stored safely in confidential conditions. A data management and sharing plan will be prepared for the trial and will include reference to confidentiality, access and security arrangements. All trial-specific documents, except for the signed consent form and follow-up contact details, will refer to the participant with a unique study participant number/code and not by name. Trial data will be collected and managed using REDCap (Research Electronic Data Capture) electronic data capture tools hosted at the Oxford Clinical Trials Research Unit, University of Oxford. REDCap is a secure, web-based application.

### Sample size

The main feasibility objective and therefore the basis of the sample size estimate is participant recruitment from at least five centres with a staggered start. The target sample size is 84 participants, equivalent of one to two participants per month per site over 12 months, allowing for staggered opening of sites. Seventy is the recommended minimum target sample size when including an estimate of the SD in an external pilot trial ^39^. The sample size has been increased from 70 to a total of 84 participants in order to increase precision of the estimate of the standard deviation of SPADI at 3 months, the proposed primary outcome for the definitive trial, and to take into account possible attrition (based on an attrition rate of 15%). This attrition rate is a conservative estimate based on attrition rates for the GRASP (14% at six months) ^33^ and UK FROST (11% at three months) ^9^ trials, which represent similar populations to those anticipated in Anti-FREAZE-F. This sample size will enable an estimate of a participation rate of 30% with a precision of +/- 5% based on 280 eligible participants being identified. We have selected a conservative recruitment rate given the uncertainty around the ability to identify, recruit and inject participants with early stage frozen shoulder.

### Statistical methods

Feasibility outcomes will be reported, including the number of participants approached, those eligible, consenting to randomisation and follow-up, the delivery of intervention (including time to first injection), withdrawal rate, the number receiving a second injection (per group and overall) and data completeness. Baseline characteristics (including possible stratification factors for the definitive trial) will be reported using descriptive statistics, separately per group and overall either using the mean and SD or median and inter-quartile range (if not normally distributed) for continuous variables and the number and percentage of participants in each group for categorical variables. All statistical analyses will be carried out using Stata (current version). Measures of central location and dispersion of clinical and patient reported outcome measures, including health resource use, at three months will be reported and differences between treatments for the intention-to-treat population (i.e., the population of participants as randomised) will be reported with 95% confidence intervals. The SD of the proposed primary outcome, SPADI at three months, will be used to inform an estimation of the sample size required for the definitive trial. An estimate of treatment effectiveness will be reported together with the 95% confidence interval. This along with change from baseline in range of shoulder motion at three months will provide an indication of potential efficacy of the intervention but will not be powered to provide a definitive result, which will only follow the fully powered definitive main trial. Even small increases in range of shoulder motion are important to people with frozen shoulder, where pain and restricted shoulder movement can be very debilitating. Therefore, we will use a 10-degree improvement in active flexion with any associated improvement in active external rotation of the shoulder to mean a potentially important difference ^40^. If we observe this difference between groups in our feasibility study with short follow up and a small sample this would indicate potential efficacy, which should then be formally, tested in the full trial. Adverse events graded 3 or above will be summarised per group and overall in all participants.

### Embedded qualitative study

The aims of the qualitative sub-study are to explore the participants’ experiences of being recruited to a randomised trial of anti-TNF injection for frozen shoulder, of the treatment received and follow-up schedule, and to understand what helps participant recruitment to the trial intervention. We will interview a purposive sample of up to 15 participants (or until we reach data saturation) to provide variability for age, gender, ethnicity and geographical representation. Participants will be invited to receive further information about the qualitative sub-study at the time of consent to the main trial. Individual telephone interviews will be conducted within four weeks of either intervention delivery by a qualitative researcher blinded to the intervention) using a semi-structured interview guide with open-ended questions.

### Progression criteria

Progression criteria for the future definitive trial will be judged using a traffic light system whereby ‘Green’ indicates it is feasible to proceed to a definitive trial with the current procedures, ‘Amber’ indicates modification to one or more aspect of the study is required before proceeding to the future definitive trial, and ‘Red’ indicates it is not feasible ^41^. The decision to progress to a future definitive trial will be made by the Trial Management Group in conjunction with the Trial Oversight Committee, based on the criteria in Table 3. It will also be informed by findings from the embedded qualitative sub-study and any potential signal of efficacy with improvements in range of shoulder motion as a result of anti-TNF injection.

**Table 3.** Progression criteria.

	Green	Amber	Red
Feasibility to recruit: % of potentially eligible patients with frozen shoulder screened across 5 sites in 12 months eligible for recruitment	≥33%	≥20% to 32%	<20%
Success of consent process: % of eligible participants consented	≥33%	≥20% to 32%	<20%
Intervention delivery: % of participants receive 1 ^st^ injection as randomised within specified timeframe	≥75%	≥50% to 74%	<50%

## Ethics and dissemination

Ethics approval was obtained from the Newcastle North Tyneside 1 Research Ethics Committee (REC: 21/NE/0214) (20/12/2021), approved by the UK Competent Authority, the Medicines and Healthcare Regulatory Agency (MHRA) (EudraCT: 2021-003509-23) and prospectively registered (ISRCTN: 27075727; ClinicalTrials.gov: NCT05299242). A Combined Trial Oversight Committee has been appointed to independently monitor progress of the trial and recommend whether there are any ethical or safety reasons why the trial should not continue. Summary results data will be included on the trial registration database within 12 months of the end of the trial. Requests for data (anonymised trial participant level data) will only be provided to external researchers who provide a methodologically sound proposal to the trial team (and who will be required to sign a data sharing access agreement with the Sponsor [University of Oxford]) and/or in accordance with funder guidance. The trial results will be published in an open-access journal, in accordance with funder policy on open-access research. The trial results will be reported following the Consolidated Standards of Reporting Trials (CONSORT) guidelines. We will inform participants of the results of trial feasibility criteria. The participants will be asked if they would like to be informed of this as part of the consent process.

**Study status:** Not yet open to recruitment.
