# Supplementary material for: Anti-TNF (adalimumab) injection for the treatment of adults with frozen shoulder during the pain predominant stage protocol for a multi-centre, randomised, double blind, parallel group, feasibility trial
Source: NIHR Open Res. 2023 Apr 24;2:28. Originally published 2022 Apr 12. [Version 2] doi: 10.3310/nihropenres.13275.2 (PMC10593336; doi:10.3310/nihropenres.13275.2)
Supplement: Peer review history

# Reviewer report: Reviewer response for version 2

**DOI:** 10.3310/nihropenres.14565.r29197

**Published:** 2023-04-26

**Authors:** Matthew Page

**Affiliations:** 1 School of Public Health and Preventive Medicine, Monash University, Melbourne, Vic, Australia

I have no further comments to make.

Is the study design appropriate for the research question?

Yes

Is the rationale for, and objectives of, the study clearly described?

Yes

Are sufficient details of the methods provided to allow replication by others?

Yes

Are the datasets clearly presented in a useable and accessible format?

Not applicable

Reviewer Expertise:

Clinical trial methodology; Shoulder pain

I confirm that I have read this submission and believe that I have an appropriate level of expertise to confirm that it is of an acceptable scientific standard.

# Reviewer report: Reviewer response for version 2

**DOI:** 10.3310/nihropenres.14565.r29198

**Published:** 2023-04-24

**Authors:** Stefan Siebert

**Affiliations:** 1 School of Infection and Immunity, University of Glasgow, Glasgow, Scotland, UK

Thank you for the amendments/clarifications.

Is the study design appropriate for the research question?

Yes

Is the rationale for, and objectives of, the study clearly described?

Yes

Are sufficient details of the methods provided to allow replication by others?

Partly

Are the datasets clearly presented in a useable and accessible format?

Not applicable

Reviewer Expertise:

Rheumatology; biologics including TNF inhibitors; clinical trials.

I confirm that I have read this submission and believe that I have an appropriate level of expertise to confirm that it is of an acceptable scientific standard.

# Reviewer report: Reviewer response for version 1

**DOI:** 10.3310/nihropenres.14394.r29120

**Published:** 2023-04-03

**Authors:** Stefan Siebert

**Affiliations:** 1 School of Infection and Immunity, University of Glasgow, Glasgow, Scotland, UK

The authors present a protocol for multi-centre, randomised, double-blind feasibility study of intra-adalimumab adalimumab injections for early frozen shoulder. The protocol is very well written, with clear justification and methods that address the primary objectives.

 Importantly for a feasibility study, the progression criteria for the main study are clearly pre-defined.

 I have a few comments, relating to some aspects where the protocol is slightly unclear or not specific:

 1. Timing of follow-up/final outcome and Table 2: The final assessment is at 3 months but it appears from Table 2 that this is 3 months from screening rather than first injection. I would have expected the latter in light of the variable timing from screening to 1 ^st^ injection (meaning some participants may only have 10 weeks from injection) and this may be what is intended, although I note assessments are only done at screening. Either way, the authors should clearly indicate the timepoint for screening in order for others to be able to replicate this work: i.e. either [“Screening week 0”] or [“Screening week -2 to 0” and “1 ^st^ injection = week 0”].

 It would also be more typical and helpful to express this final outcome timing in weeks (i.e. 12 weeks) rather than months in order to avoid potential confusion relating to whether this is 3 calendar months or 12 weeks.

 2. It is also unclear from the protocol what would occur if >2 weeks passed from screening to 1 ^st^ injection. This is particularly import as ITT analysis based on randomization so need to know if would be withdrawn, rerandomized etc. The same applies to what would occur if >3 weeks between 1 ^st^ and 2 ^nd^ injection visit – I assume no injection would be given but actions for both scenarios should be clearly stated.

 3. As a CT-IMP, I would have expected standard adverse event reporting requirements to apply.

 4. Eligibility criteria – timing from symptom onset: It is unusual to include an “approximate” cut-off in inclusion criteria as this introduces potential variability, both within this study and with replication studies. I presume this was included to facilitate recruitment, and may be acceptable for this feasibility study (provided clearly and rigorously recorded), but it is largely meaningless without some upper limit (is 4 months ok, what about 5 months?) and would not be acceptable for the main study.

 5. Eligibility criteria – frozen shoulder diagnosis: I found the inclusion criteria slightly vague and open to interpretation. I would have expected more formal diagnostic criteria for frozen shoulder for an RCT. While I accept this study may be intended to reflect real-world-practice, if the authors propose to use results from this and/or the subsequent main study to obtain approvals for this treatment (adalimumab is not currently licensed for intra-articular injection), I would recommend early discussions with the relevant regulatory bodies to ensure they would accept these criteria for licensing purposes.

 I look forward to seeing the results of this important study in the future.

Is the study design appropriate for the research question?

Yes

Is the rationale for, and objectives of, the study clearly described?

Yes

Are sufficient details of the methods provided to allow replication by others?

Partly

Are the datasets clearly presented in a useable and accessible format?

Not applicable

Reviewer Expertise:

Rheumatology; biologics including TNF inhibitors; clinical trials.

I confirm that I have read this submission and believe that I have an appropriate level of expertise to confirm that it is of an acceptable scientific standard.

# Response

**Published:** 2023-04-14

**Authors:** Sally Hopewell

**Affiliations:** University of Oxford, UK

Thank you for the positive comments and suggestions for improving the protocol for the Anti-Freaze-F trial. We have included our response to each of the reviewer comments below:

**Reviewer Report 2:**

*The authors present a protocol for multi-centre, randomised, double-blind feasibility study of intra-adalimumab adalimumab injections for early frozen shoulder. The protocol is very well written, with clear justification and methods that address the primary objectives. Importantly for a feasibility study, the progression criteria for the main study are clearly pre-defined. I have a few comments, relating to some aspects where the protocol is slightly unclear or not specific:*

*1. Timing of follow-up/final outcome and Table 2: The final assessment is at 3 months but it appears from Table 2 that this is 3 months from screening rather than first injection. I would have expected the latter in light of the variable timing from screening to 1 ^st^ injection (meaning some participants may only have 10 weeks from injection) and this may be what is intended, although I note assessments are only done at screening. Either way, the authors should clearly indicate the timepoint for screening in order for others to be able to replicate this work: i.e. either [“Screening week 0”] or [“Screening week -2 to 0” and “1 ^st^ injection = week 0”].*

 RESPONSE: Timing of follow up/final outcome is at 3 months (i.e. 12 weeks) post randomisation. We have clarified this through the protocol.

*It would also be more typical and helpful to express this final outcome timing in weeks (i.e. 12 weeks) rather than months in order to avoid potential confusion relating to whether this is 3 calendar months or 12 weeks.*

 RESPONSE:  Reporting of the outcome measures is at 3 months (i.e. 12 weeks). We have clarified this in the protocol.

*2. It is also unclear from the protocol what would occur if >2 weeks passed from screening to 1 ^st^ injection. This is particularly import as ITT analysis based on randomization so need to know if would be withdrawn, rerandomized etc. The same applies to what would occur if >3 weeks between 1 ^st^ and 2 ^nd^ injection visit – I assume no injection would be given but actions for both scenarios should be clearly stated.*

 RESPONSE: This is a feasibility study and so one of our main feasibility objectives is to assess the practicalities for trial sites to deliver the intervention, including the time to first injection and number of participants receiving second injection. This information would then be used to inform any future definitive trial. Therefore, we would respectfully submit that the issue of ITT analysis is not applicable here.

*3. As a CT-IMP, I would have expected standard adverse event reporting requirements to apply.*

 RESPONSE: Procedures for recording adverse events are described in the protocol in the section “Adverse events”. We will record clinician assessed adverse events, graded 3 and above, occurring during the trial for each participant, from their consent until the three month (i.e. 12 weeks) follow up and that are considered related to the trial medication (adalimumab/placebo). 

*4. Eligibility criteria – timing from symptom onset: It is unusual to include an “approximate” cut-off in inclusion criteria as this introduces potential variability, both within this study and with replication studies. I presume this was included to facilitate recruitment, and may be acceptable for this feasibility study (provided clearly and rigorously recorded), but it is largely meaningless without some upper limit (is 4 months ok, what about 5 months?) and would not be acceptable for the main study.*

 RESPONSE: Recruitment will be based on the participant’s predominant symptom being pain rather than limitation of movement or function. The duration of the pain-predominant phase can be very variable, usually between 3 – 9 months and, therefore it is difficult to precisely stipulate inclusion based on a precise time period. We have now clarified this in the protocol.

*5. Eligibility criteria – frozen shoulder diagnosis: I found the inclusion criteria slightly vague and open to interpretation. I would have expected more formal diagnostic criteria for frozen shoulder for an RCT. While I accept this study may be intended to reflect real-world-practice, if the authors propose to use results from this and/or the subsequent main study to obtain approvals for this treatment (adalimumab is not currently licensed for intra-articular injection), I would recommend early discussions with the relevant regulatory bodies to ensure they would accept these criteria for licensing purposes.*

 RESPONSE: We used the BESS (British Elbow and Shoulder Society) criteria for diagnosis of pain predominant early phase frozen shoulder, which is widely used by physiotherapists and shoulder specialists within the NHS. In the protocol we state that imaging, including plain radiographs, may be used to confirm the diagnosis of frozen shoulder and rule out other pathology such as glenohumeral arthritis as would happen as per standard NHS care (i.e. not as part of trial procedures). Regulatory approval from the MHRA was successfully obtained for the trial.

# Reviewer report: Reviewer response for version 1

**DOI:** 10.3310/nihropenres.14394.r29073

**Published:** 2023-03-21

**Authors:** Matthew Page

**Affiliations:** 1 School of Public Health and Preventive Medicine, Monash University, Melbourne, Vic, Australia

Hopewell *et al.* have prepared a clear and compelling protocol for a study assessing the feasibility of conducting a large randomised controlled trial to assess whether intra-articular injection of anti-TNF (adalimumab) can reduce pain and improve function in people with pain predominant early stage frozen shoulder. The methods proposed are rigorous and the protocol is reported in enough detail to allow others to replicate the planned methods. I have only a few minor suggestions.

**Name of the trial:** This is extremely minor, but every time I read “Anti-Freaze-F trial” I wondered why it was not called the “Anti-Freeze-F trial”. Perhaps a brief explanation for the name would help.

**Plain language summary:** Overall, this section is very clear and informative. However, I think there needs to be a clearer link between the first and second paragraph. The first paragraph ends with the claim that there is no evidence that current treatments, including rest, painkillers, anti-inflammatories, physiotherapy and steroid injections lead to significant benefit in the long term, and that steroid injections only help in the short term. Then in the second paragraph the authors claim that the aim of the study is to find out if it is feasible to “run a larger trial to test whether an injection of adalimumab can reduce pain and prevent the disease from getting worse”. However, it’s not clear why the authors are investigating the effects of adalimumab, nor what adalimumab even is (is it a type of one of the interventions described as ineffective in the first paragraph, or something else entirely?).

*Postscript:* Oh, I see a rationale for the choice of adalimumab appears at the end of the third paragraph. Hmm, I think it would flow better if this text were moved earlier.

**Eligibility:** By specifying that “Men and women aged 18 years and above” were eligible for this study, does this mean that non-binary people and those who do not identify with either of those genders are ineligible? If not, perhaps it's more inclusive to just write “People aged 18 years and above”.

**Figure 1:** It might help to clarify that the second injections will be delivered 2-3 weeks after the first injection. It would also help to specify if follow-up is at 3-months from randomisation, or 3-months after receiving the second injection.

**Informed consent, baseline assessment and trial specific screening tests:** It is not immediately clear to me why “Participants will undergo serological testing to check for latent Tuberculosis (TB) and Hepatitis B surface antigen”.

**Table 2:** Love this table, really helpful!

**Outcomes:** The authors claim that “The choice of outcome measures is based on Outcome Measures in Rheumatology (OMERACT) 2016 core outcome set for shoulder disorder”. However, the core outcome set cited is a preliminary core outcome set, which was finalised and endorsed by OMERACT in 2019. So I suggest the final core outcome set be cited - Ramiro *et al.* (2019) ^1^.

 Good luck with the study!

Is the study design appropriate for the research question?

Yes

Is the rationale for, and objectives of, the study clearly described?

Yes

Are sufficient details of the methods provided to allow replication by others?

Yes

Are the datasets clearly presented in a useable and accessible format?

Not applicable

Reviewer Expertise:

Shoulder pain; Trial methodology; Systematic review methodology

I confirm that I have read this submission and believe that I have an appropriate level of expertise to confirm that it is of an acceptable scientific standard.

## References

[1] : The OMERACT Core Domain Set for Clinical Trials of Shoulder Disorders. *J Rheumatol* .2019;46(8) : 10.3899/jrheum.181070 969-975 10.3899/jrheum.181070 30709947

# Response

**Published:** 2023-04-14

**Authors:** Sally Hopewell

**Affiliations:** University of Oxford, UK

Thank you for the positive comments and suggestions for improving the protocol for the Anti-Freaze-F trial. We have included our response to each of the reviewer comments below:

**Reviewer Report 1:**

*Hopewell et al. have prepared a clear and compelling protocol for a study assessing the feasibility of conducting a large randomised controlled trial to assess whether intra-articular injection of anti-TNF (adalimumab) can reduce pain and improve function in people with pain predominant early stage frozen shoulder. The methods proposed are rigorous and the protocol is reported in enough detail to allow others to replicate the planned methods. I have only a few minor suggestions.*

*Name of the trial: This is extremely minor, but every time I read “Anti-Freaze-F trial” I wondered why it was not called the “Anti-Freeze-F trial”. Perhaps a brief explanation for the name would help.*

 RESPONSE: The acronym is derived from ANTI-tnF for the tREAtment of froZEn shoulder – Feasibility

*Plain language summary: Overall, this section is very clear and informative. However, I think there needs to be a clearer link between the first and second paragraph. The first paragraph ends with the claim that there is no evidence that current treatments, including rest, painkillers, anti-inflammatories, physiotherapy and steroid injections lead to significant benefit in the long term, and that steroid injections only help in the short term. Then in the second paragraph the authors claim that the aim of the study is to find out if it is feasible to “run a larger trial to test whether an injection of adalimumab can reduce pain and prevent the disease from getting worse”. However, it’s not clear why the authors are investigating the effects of adalimumab, nor what adalimumab even is (is it a type of one of the interventions described as ineffective in the first paragraph, or something else entirely?). Postscript: Oh, I see a rationale for the choice of adalimumab appears at the end of the third paragraph. Hmm, I think it would flow better if this text were moved earlier.*

 RESPONSE: We have now added the explanation for the use of adalimumab before the description of the trial.

*Eligibility: By specifying that “Men and women aged 18 years and above” were eligible for this study, does this mean that non-binary people and those who do not identify with either of those genders are ineligible? If not, perhaps it's more inclusive to just write “People aged 18 years and above”.*

 RESPONSE: We have reworded this as suggested to be more inclusive.

*Figure 1: It might help to clarify that the second injections will be delivered 2-3 weeks after the first injection. It would also help to specify if follow-up is at 3-months from randomisation, or 3-months after receiving the second injection.*

 RESPONSE: We have changed Figure 1 to clarify the second injection is given 2-3 weeks after the first injection. We have also clarified that follow up is 3 months (i.e. 12 weeks) post-randomisation.

*Informed consent, baseline assessment and trial specific screening tests: It is not immediately clear to me why “Participants will undergo serological testing to check for latent Tuberculosis (TB) and Hepatitis B surface antigen”.*

 RESPONSE: Continuous administration of anti-TNF is associated with reactivation of latent TB or hepatitis B. Whilst the risk is extremely low following a single injection, to ensure participant safety we specified that participants will undergo serological testing before the second injection. Those testing positive will be referred to their local infectious diseases service and will not receive the second injection

*Table 2: Love this table, really helpful!*

 RESPONSE: Thank you!

*Outcomes: The authors claim that “The choice of outcome measures is based on Outcome Measures in Rheumatology (OMERACT) 2016 core outcome set for shoulder disorder”. However, the core outcome set cited is a preliminary core outcome set, which was finalised and endorsed by OMERACT in 2019. So I suggest the final core outcome set be cited - Ramiro et al. (2019)* *^1^* *.*

 RESPONSE: We have updated this reference.
